# Phenolic Content and Antioxidant Activity of *Hibiscus cannabinus* L*.* Seed Extracts after Sequential Solvent Extraction

**DOI:** 10.3390/molecules171112612

**Published:** 2012-10-25

**Authors:** Noordin Mohd Yusri, Kim Wei Chan, Shahid Iqbal, Maznah Ismail

**Affiliations:** 1 Laboratory of Molecular Biomedicine, Institute of Bioscience, Universiti Putra Malaysia, 43400 UPM Serdang, Selangor, Malaysia; Email: yus_enigma@hotmail.com (N.M.Y.); chankw@ibs.upm.edu.my (K.W.C.); ranashahid313@gmail.com (S.I.); 2 Department of Nutrition and Dietetics, Faculty of Medicine and Health Sciences, Universiti Putra Malaysia, 43400 UPM Serdang, Selangor, Malaysia; 3 Department of Chemistry, University of Sargodha, Sargodha-40100, Pakistan

**Keywords:** kenaf seeds, antioxidant activity, sequential solvent extraction, phenolic content, *Hibiscus cannabinus* L.

## Abstract

A sequential solvent extraction scheme was employed for the extraction of antioxidant compounds from kenaf (*Hibiscus cannabinus* L.) seeds. Yield of extracts varied widely among the solvents and was the highest for hexane extract (16.6% based on dry weight basis), while water extract exhibited the highest total phenolic content (18.78 mg GAE/g extract), total flavonoid content (2.49 mg RE/g extract), and antioxidant activities (*p* < 0.05). DPPH and hydroxyl radical scavenging, β-carotene bleaching, metal chelating activity, ferric thiocyanate and thiobarbituric acid reactive substances assays were employed to comprehensively assess the antioxidant potential of different solvent extracts prepared sequentially. Besides water, methanolic extract also exhibited high retardation towards the formation of hydroperoxides and thiobarbituric acid reactive substances in the total antioxidant activity tests (*p* < 0.05). As conclusion, water and methanol extracts of kenaf seed may potentially serve as new sources of antioxidants for food and nutraceutical applications.

## 1. Introduction

A number of epidemiological studies have demonstrated that many herbs and medicinal plants have potential disease-preventive effects against oxidative stress [1,2], and a significant number of herbs, spices, cereals, and legumes have been explored as potential sources of antioxidants. Besides the disease-preventive and health-promoting effects of these natural sources of antioxidants, they have profound effects on food preservation, *i.e*., degradation against oxidative deterioration. 

Lipid oxidation is one of the causes of deterioration that occurs in food products during processing and storage [3]. It badly affects sensory attributes of food products by imparting undesirable rancid flavors, textures and tastes to food products [4], resulting in an overall decrease in the shelf-life of foods. Besides this, lipid oxidation will also jeopardize the nutritional quality of the food products by interfering with the absorption/bioavailability of macronutrients (such as proteins or folic acid) and accelerate the development of a few oxidative stress related diseases, such as atherosclerosis, cancers and Alzheimer’s disease [3]. Owing to that, it is therefore important to minimize the process of lipid oxidation. 

Synthetic antioxidants such as BHA and BHT have been being commonly used during food processing in order to prolong the storage stability of fats, oils and lipid-containing foods [3], but involvement of these synthetic antioxidants in a number of physiological disorders and diseases has been reported. Owing to that, natural antioxidants such as phenolic compounds and other phytochemicals have emerged as safer alternatives to synthetic antioxidants in recent years. According to Miraliakbari and Shahidi [5], natural antioxidants can function as free radical scavengers, reducing agents, chelators of prooxidant metals, or as quenchers of singlet oxygen and thus delay the lipid oxidation process in food products. A number of antioxidative sources based on natural origin have been explored so far, but still there exists need for the search of newer sources, which may be safer, more economical and preferably from dietary sources. This study is part of that series of investigations.

Kenaf (*Hibiscus cannabinus* L.) is an annual herbaceous plant that has great potential as a source of fiber, energy, and feedstock. It has been cultivated as a commodity plant in many countries such as China, India, and Thailand. Currently, Malaysia has started to cultivate kenaf as its fourth industrial crop in order to replace tobacco plantations and research on kenaf seed production was initiated in Malaysia in early 2000 [6]. Kenaf seed is a byproduct of the kenaf industry. Previous studies have suggested that kenaf seed oil (23.7%) is suitable for human consumption due to its unique fatty acid composition and antioxidant activity [7,8]. Nevertheless, studies on the phenolic content and antioxidant activity of different kenaf seed extracts are very limited at present. Thus, this study was initiated in order to investigate the phenolic content and antioxidant activity of kenaf seed extracts, obtained through sequential solvent extraction.

## 2. Results and Discussion

### 2.1. Yield of Sequentially Prepared Extracts, Total Phenolic Content and Total Flavonoid Content

Extracts of kenaf seeds were obtained following a sequential solvent extraction procedure and yield of extract obtained for each solvent was calculated separately (Table 1). Yields varied over a wide range of 4.63%–16.60% (based on dry weight basis) among the solvents. The highest yield was obtained for hexane extract (16.60%) followed by water (6.4%), methanol (4.7%) and chloroform (4.6%), respectively; differences being significant (*p* < 0.05) among the solvents. Such a wide variation in the yield of extracts is due to the different polarities of the extraction solvents. The high extraction yield for hexane is due to the high content of oil in kenaf seed [9]; ranging from 21.4% to 26.4% [7,8,10].

**Table 1:** Yield of sequentially extracted kenaf seeds; total phenolic and total flavonoid contents.

Extract (according to polarity)	Yield (% w/dw sample)	Total Phenolic Content (mg GAE/g extract)	Total Flavonoids (mg RE/g extract)
Water	6.65 ± 0.07 ^a^	18.78 ± 0.35 ^a^	2.49 ± 0.53 ^a,b^
Methanol	4.66 ± 0.20 ^b^	5.36 ± 0.88 ^b^	1.77 ± 0.28 ^a^
Chloroform	4.63 ± 0.72 ^b^	12.26 ± 1.27 ^c^	2.94 ± 0.10 ^b^
Hexane	16.60 ± 0.18 ^c^	2.16 ± 0.21 ^d^	1.64 ± 0.38 ^a^

Note: Data are expressed as mean ± SD; Values with different superscripts within the same column are significantly (*p* < 0.05) different and *vice versa*.

Total phenolic contents (TPC) for kenaf seed extracts, obtained sequentially in different solvents, were determined spectrophotometrically using gallic acid as calibration standard. The TPC values varied over a wide range, *i.e*., 2.16–18.78 mg GAE/g, among the extracts prepared in different solvents (Table 1).

Highest TPC (18.78 mg GAE/g extract) was observed for aqueous extract with significant differences for TPC in the extracts of other solvents (*p* < 0.05). The findings reveal that most of the phenolic compounds in kenaf seeds are highly polar in nature, and thus more efficiently extractable by polar solvents. The highest yield in water may be attributed to the chemical structure of phenolic compounds, which contain one or more hydrophilic hydroxyl groups. Moreover, the findings are in agreement with the observations of Matthaus [11], who reported the high efficiency of polar solvents, *i.e*., water and methanol, in extracting phenolic compounds from several oilseeds. 

Total flavonoid contents ranged over 1.64–2.94 mg RE/g extract. Highest TFC was recorded for chloroform extract, while the lowest was for hexane extract; differences again being significant for TFC among the extracts prepared in different solvents (*p* < 0.05). Total flavonoid content is well correlated with TPC with ‘r’ = 0.78, indicating that flavonoids might be the major contributors towards the phenolic compounds count for kenaf seeds. Order of TPC and TFC was quite opposite to the order of yields obtained.

### 2.2. Antioxidant Activity Tests

Table 2 shows DPPH and hydroxyl radical scavenging, beta-carotene bleaching inhibitory and metal chelating activities of kenaf seed extracts prepared sequentially in different solvents. The DPPH radical scavenging activity of the extracts ranged from 0.40 to 2.30 µg ATE/g extract, and in the order hexane < chloroform < methanol < water (*p* < 0.05). On the other hand, hydroxyl radical scavenging activity ranged from 3.33 to 23.64 g DMSO/g extract and increased in the order: hexane extract < methanol extract < chloroform extract < water extract (*p* < 0.05). 

**Table 2:** Antioxidant activities of kenaf seed extracts.

Kenaf seed extracts	DPPH Radical scavenging activity (µg ATE/g sample)	Hydroxyl radical scavenging activity (g DMSOE/g sample)	β-Carotene Bleaching (mg ATE/g extract)	Metal Chelating (mg CAE/g extract)
Water	2.30 ^a^	23.64 ^a^	7.15 ^a^	81.62 ^a^
Methanol	0.62 ^b^	20.30 ^b^	1.42 ^b^	43.27 ^bc^
Chloroform	0.56 ^b^	23.48 ^a^	1.46 ^b^	54.45 ^b^
Hexane	0.40 ^c^	3.33 ^c^	0.71 ^c^	29.86 ^c^

Note: Data is expressed in mean ± SD; Value with different superscripts within the same column are statistically significant (*p* < 0.05).

Beta-carotene bleaching inhibitory activity of the extracts was determined from the linear regression equation; y = 7.9982x + 6.511 (*r*^2^ = 0.9425), and it ranged over 0.71 to 7.15 mg ATE/g extract and decreased in the order: water extract > chloroform extract > methanol extract > hexane extract (*p* < 0.05). 

Metal chelating activity determined from the linear regression equation y = 0.1666x + 2.1005 (*r*^2^ = 0.9935), ranged from 29.86 to 81.62 mg CAE/g sample and is given as: water extract > chloroform extract > methanol extract > hexane extract (*p* < 0.05).

According to these results, water extract exhibited the highest antioxidant activity as compared to other solvent extracts (*p* < 0.05), demonstrating that water extract might possess higher levels of primary antioxidant compounds that are capable of effectively scavenging free radicals and neutralizing hydroperoxides that are responsible for discoloration of β-carotene. Besides this, the results also show that water extract possesses the highest metal chelating activity; a secondary antioxidant mechanism that inhibits prooxidant transition metals from catalyzing the breakdown of hydroperoxides.

**Figure 1:**
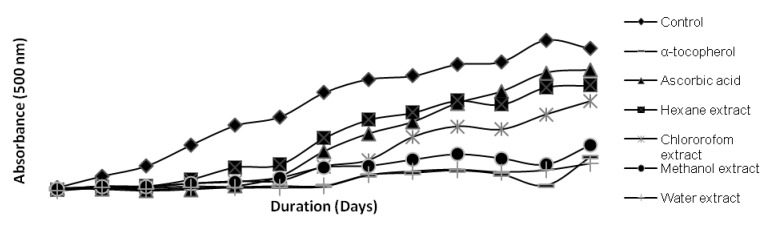
Antioxidant activity of kenaf seed extracts determined by FTC assay.

The FTC test measures the amount of peroxides during the initial stages of lipid peroxidation. Thus, a low absorbance value is indicative of high antioxidant activity/hydroperoxide inhibitory activity of tested sample. Figure 1 shows the hydroperoxides inhibitory activity of kenaf seed extracts throughout the 13 days of incubation period. 

Inhibitory activities of the tested samples (on the twelfth day) are arranged in the following descending order: α-tocopherol > water extract > methanolic extract > chloroform extract > hexane extract ≥ ascorbic acid (*p* < 0.05). Water and methanolic extracts exhibited the highest hydroperoxides inhibitory activity, which is slightly lower than that of α-tocopherol, but higher than that of ascorbic acid and other tested kenaf seed extracts (*p* < 0.05). 

Results from the FTC test confirm earlier findings of this study, indicating that kenaf seed water and methanol extracts might possess high levels of primary antioxidant compounds that are capable of suppressing hydroperoxide formation during the initial stages of lipid peroxidation through radical-chain breaking mechanisms. Furthermore, HPLC analysis (unpublished data) conducted in our laboratory showed that the aqueous-methanolic extract from defatted kenaf seed cake contains predominantly primary antioxidant compounds such as gallic acid, (+)-catechin, chlorogenic acid, hydroxybenzoic acid, syringic acid and vanillin, which also supports the findings of the FTC test.

**Figure 2:**
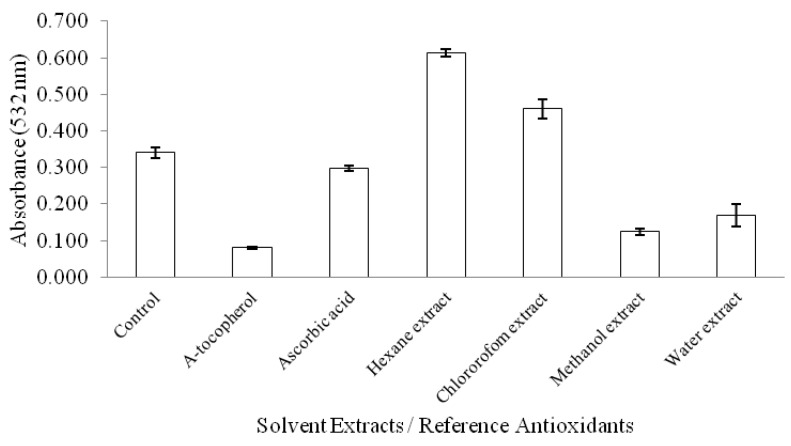
Thiobarbituric acid reactive substances measured for kenaf seed extracts fractionated in different solvents.

Figure 2 shows TBARS inhibitory activity of the kenaf seed extracts. Overall, water and methanolic extracts exhibited the highest inhibitory activity towards the formation of TBARS in the following descending order: methanol > water > chloroform ≥ hexane (*p* < 0.05). Hexane and chloroform extracts showed higher absorbance (*p* < 0.05) than control, indicating that both the extracts might contain prooxidative compounds that induce the breakdown of hydroperoxides and amplify the generation of TBARS. The lower TBARS values exhibited by aqueous and methanolic extracts are probably due to the least hydroperoxides accumulation in both samples, as discussed in the FTC test. Secondary antioxidative compounds also play an important role in inhibiting the pro-oxidant transition metals from catalyzing the breakdown of hydroperoxides to TBARS. As reported earlier, water extract of kenaf seeds possesses high metal chelating activity towards prooxidative iron, thus supporting the findings of the TBARS assay. Order of antioxidant activity obtained on the basis of different assays is also opposite to order of extract yield obtained.

## 3. Experimental

### 3.1. Plant Material

Kenaf seeds (variety V 36) were provided by the National Kenaf Tobacco Board Malaysia, Pasir Putih, Kelantan, Malaysia.

### 3.2. Chemicals and Reagents

All the solvents and chemicals used included: hexane, chloroform, methanol, acetonitrile, ethanol and trichloroacetic acid (Fisher Scientific, Leicestershire, UK); Folin-Ciocalteu reagent (Fluka Biochimica, Buchs, Switzerland); NH_4_SCN 99.99% and linoleic acid (Sigma Chemical Co., St. Louis, MO, USA); HCl 37% (Merck KGaA, Darmstadt, Germany); FeCl_2_-80 mesh 98%, L-ascorbic acid 99%, EDTA, 1,1-diphenyl-2-picrylhydrazyl (DPPH), AlCl_3_, 5,5-dimethylpyrroline-*N*-oxide (DMPO), dimethyl sulphoxide (DMSO), rutin, α-tocopherol (Sigma-Aldrich, Munich, Germany); thiobarbituric acid (AppliChem GmbH, Darmstadt, Germany), phosphate buffer solution pH 7.00 ± 0.02, 20 °C (R&M Chemicals, Bristol, UK) and gallic acid (Sigma-Aldrich, Madrid, Spain), were of analytical grade. 

### 3.3. Preparation of Kenaf Seeds

Approximately 200 g of kenaf seeds were weighed and cleaned thoroughly in a sonicator bath (Hwasin Technology, Seoul, Korea) for 1 h. Then, the cleaned kenaf seeds were dried in an oven at 40 °C until a constant weight was attained. Finally, dried kenaf seeds were stored at 4 °C prior to further analyses.

### 3.4. Sequential Solvent Extraction of Kenaf Seeds

**Figure 3:**
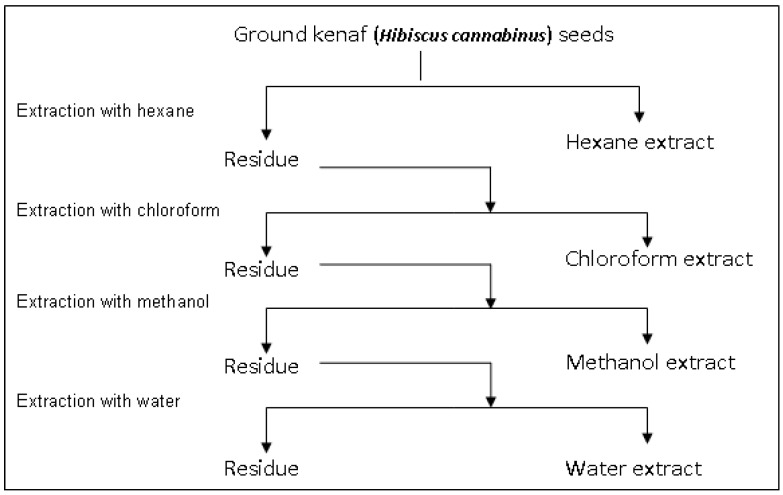
Sequential extraction of kenaf (*Hibiscus cannabinus*) seeds.

In brief, dried kenaf seeds (90 g) were ground to fine powder using a stainless steel blender (Waring Commercial, Torrington, CT, USA) for 1 min followed by mixing with 360 mL hexane, homogenizzation (Ultra-Turrax, Staufen, Germany) at 13,000 rpm for 15 min and sonication (Hwasin Technology, Seoul, Korea) up to 1 h. The resulting mixture was filtered through Whatman No 1 filter paper and hexane was removed from the filtrate under reduced pressure with a rotatory evaporator (Büchi, Flawil, Switzerland). The residue was further extracted with chloroform, methanol, and water sequentially in a serial manner (Figure 3). Finally, each extract was weighed and the yield was calculated. The kenaf seed extracts were kept at −80 °C prior to further analyses.

### 3.5. Total Phenolic Content (TPC)

Poor solubility of hexane and chloroform extracts in aqueous solutions limited the measurement of TPC. Therefore, hexane and chloroform extracts were purified [12] prior to TPC determination. Total phenolic contents of kenaf seed extracts were determined according to the method of Iqbal *et al.* [13]. In brief, individual extract (10 mg) was dissolved in methanol (1 mL). Out of these aliquots, 0.5 mL of each were transferred to separate flasks, Folin-Ciocalteu reagent (2.5 mL) was added to each, followed by addition of sodium carbonate solution (2 mL; 7.5%). The mixtures were stirred well and incubated at 40 °C for 30 min to ensure the completion of reaction. Finally, the absorbance of resulting mixtures was read at 765 nm using spectrophotometer (Pharmaspec UV-1700, Shimadzu, Kyoto, Japan). Total phenolic content was expressed as mg gallic acid equivalents per g of extract (mg GAE/g extract).

### 3.6. Total Flavonoid Content (TFC)

Total flavonoid content of different sequentially prepared extracts was determined following a previously reported method [14] with slight modifications. Extracts (100 mg) were dissolved in DMSO (10 mL) for preparing stock solutions. Stock solution (500 µL) was mixed with equal volumes of AlCl_3_ (2%) and the mixture was left at shelf for 10 min followed by recording the absorbance spectrophotometrically (Pharmaspec UV-1700) at 435 nm. Total flavonoid content was expressed as mg rutin equivalent/g extract (mg RE/g extract). 

### 3.7. 2,2-Diphenyl-1-picrylhidrazyl (DPPH) Radical Scavenging Assay

DPPH radical scavenging activity was determined following the method of Ramadan *et al.* [12] with slight modifications. In brief, methanolic solution of DPPH radical (0.39 mL; 0.1 mM) was reacted with each kenaf seed extract (0.1 mL) of different concentration under ambient conditions. The mixtures were shaken vigorously and were allowed to stand for 60 min in the dark. Finally, DPPH radical scavenging activity was measured using electron spin resonance spectrometer (ESR, JEOL FA100, Chiba, Japan) under following conditions: modulation frequency = 100 kHz; center field = 336.734 ± 5 mT; sweep time = 1 min; power = 8 mW; mod width = 0.2 mT; time constant = 0.15 s; amplitude = 160. α-Tocopherol was used as standard in this test. DPPH radical scavenging activity of kenaf seed extracts was expressed as mg α-tocopherol equivalent (ATE)/g extract. 

### 3.8. Hydroxyl Radical Scavenging Assay

Hydroxyl radical was generated through Fenton reaction and scavenging activity of kenaf seed extracts was measured using ESR (JEOL FA100). In brief, DMPO (40 µL, 400 mM) was mixed with FeSO_4_ (37.5 µL, 0.4 mM), EDTA (112.5 µL, 0.1 mM), extract (60 µL, at various concentrations) and hydrogen peroxide (150 µL, 2 mM), respectively. After two min, the mixture was filled into a flat cell and hydroxyl radical intensity of the samples was measured according to conditions as follows: center field = 336.45 ± 5; power = 8 mW; mod width = 0.1 mT; sweep time = 2 min; time constant = 0.1 s; amplitude = 160; modulation frequency = 100 kHz. Dimethyl sulfoxide (DMSO) was used as standard and hydroxyl radical scavenging activity of extracts was expressed as g DMSO equivalent/g extract (g DMSOE/g extract).

### 3.9. β-Carotene Bleaching Assay

The β-carotene bleaching (BCB) potential of the extracts was determined following a previously reported method of Wettasinghe and Shahidi [15]. In brief, β-carotene solution (3 mL, 100 µg/mL chloroform) were added to linoleic acid (40 mg) and Tween 20 (400 mg). The mixture was shaken well before drying under a stream of nitrogen followed by addition of distilled water (100 mL) to the dried mixture to form a β-carotene/linoleic acid emulsion. About 1.5 mL of emulsion were mixed with methanol (20 µL, as control) or 5 mg sample/mL methanol followed by incubation of sample in water bath, for 1 h, at 50 °C. Absorbance of the sample was recorded at 470 nm spectrophotometrically (Pharmaspec UV-1700). Antioxidant activity (%AA) of the kenaf seed extracts was calculated using following formula:

%AA = 100(DR_c_ − DR_s_)/DR_c_

where:

DR = Degradation rate; (a/b)/60
a = Absorbance of sample at 470 nm before incubation
b = Absorbance of sample at 470 nm after incubation
DR_c_ = Degradation rate of the control sample
DR_s_ = Degradation rate of tested sample

and expressed as mg α-tocopherol equivalent (mg ATE/g extract).

### 3.10. Metal Chelating Activity

Metal chelating activity of kenaf seed extracts was determined following a previously reported method [16]. Reaction solutions, composed of kenaf seed methanolic extracts (600 μL) and 2 mM FeCl_2_ (40 μL) were activated by adding 5 mM ferrozine (80 μL). After thorough mixing, the reaction mixtures were left at room temperature for 10 min. Finally, metal chelating activity of kenaf seed extracts was determined by measuring absorbance at 562 nm spectrophotometerically (Pharmaspec UV-1700). Citric acid was used as a standard and results were expressed as mg citric acid equivalent/g extract (mg CAE/g extract).

### 3.11. Total Antioxidant Activity

#### 3.11.1. Ferric Thiocyanate (FTC) Test

Ferric thiocyanate test was conducted following a previously reported method [17]. Screw-capped vials, containing mixture of kenaf seed extracts (4 mg dissolved in 4 mL ethanol), linoleic acid solution in ethanol (4.1 mL, 2.51%), phosphate buffer (pH 7.0; 8.0 mL, 0.05 M) and water (3.9 mL) were placed in an oven at 40 °C in dark. In order to conduct FTC, 0.1 mL of the sample was pipetted into a test tube followed by addition of ethanol (9.7 mL, 75%; v/v), ammonium thiocyanate (0.1 mL, 30%) and ferrous chloride solution in 3.5% hydrochloric acid (0.1 mL, 20 mM) to the mixtures serially. Exactly 3 min after the addition of ferrous chloride, absorbance of resulting mixture was recorded spectrophotometrically at 500 nm (Pharmaspec UV-1700). FTC of samples was determined daily until the control sample reached its maximum absorbance. α-Tocopherol was used as standard in this test.

#### 3.11.2. Thiobarbituric Acid Reactive Substances (TBARS) Assay

Thiobarbituric acid reactive substances (TBARS) assay was performed continuously after the control sample in FTC test started to decline. Approximately, 2 mL of the sample solution from FTC test was combined with 20% aqueous trichloroacetic acid (1 mL) and 0.67% aqueous thiobarbituric acid (2 mL). Then the mixtures were placed in water bath, containing boiling water, for 10 min. After cooling under tap water, the samples were centrifuged at 3,000 rpm for 30 min. Finally, the absorbance of supernatants was read at 532 nm by using spectrophotometer (Pharmaspec UV-1700).

### 3.12. Statistical Analysis

The significant differences (*p* < 0.05) among the samples were identified using SPSS software (version 15) through ANOVA and Tukey tests. The correlations among TPC, TFC and antioxidant activities, *i.e*., DPPH scavenging, hydroxyl scavenging, β-carotene bleaching, metal-chelating, besides FTC and TBARS, were determined through Pearson correlation test.

## 4. Conclusions

Polarity of the extraction solvent has an enormous influence on the extraction yield and the antioxidant efficacy of kenaf seeds extracts. However, extraction yield gave a negative correlation with antioxidant activity in this study. Water extract possesses the best antioxidant activity in all the antioxidant tests as compared to other extracts. Therefore, water extract of kenaf seeds may potentially serve as alternative source of natural antioxidant for nutraceutical and functional food applications. 

## References

[1] Chan K.W., Iqbal S., Khong N.M.H., Babji A.S. (2011). Preparation of deodorized antioxidant rich extracts from 15 selected spices through optimized aqueous extraction. J. Med. Plants Res..

[2] Iqbal S., Younas U., Sirajuddin, Chan K.W., Sarfraz R.A., Uddin M.K.  (2012). Proximate composition and antioxidant potential of leaves from three varieties of mulberry (*Morus* spp.): A comparative study. Int. J. Mol. Sci..

[3] Karpinska M., Borowski J., Danowska-Oziewicz M. (2001). The use of natural antioxidants in ready-to-serve food. Food Chem..

[4] Chan K.W., Khong N.M.H., Iqbal S., Ch’ng S.E., Babji A.S. (2012). Preparation of clove buds deodorized aqueous extract (CDAE) and evaluation of its potential to improve oxidative stability of chicken meatballs in comparison to synthetic and natural food antioxidants. J. Food Qual..

[5] Miraliakbari H., Shahidi F. (2008). Antioxidant activity of minor components of tree nut oils. Food Chem..

[6] Agricultural Research and Development Institute, MARDI Proceedings of the third technical review meeting on the National Kenaf Research Project.

[7] Chan K.W., Ismail M.  (2009). Supercritical carbon dioxide fluid extraction of *Hibiscus cannabinus* L. seed oil: A potential solvent-free and high antioxidative edible oil. Food Chem..

[8] Mohamed A., Bhardwaj H., Hamama A., Webber C.  (1995). Chemical composition of kenaf (*Hibiscus cannabinus* L.) seed oil. Ind. Crops Prod..

[9] Conkerton E., Wan P., Richard O. (1995). Hexane and heptane as extraction solvents for cottonseed: A laboratory-scale study. J. Am. Oil Chem. Soc..

[10] Nyam K.L., Tan C.P., Lai O.M., Long K., Che Man Y. (2009). Physicochemical properties and bioactive compounds of selected seed oils. LWT-Food Sci. Technol..

[11] Matthaous B. (2002). Antioxidant activity of extracts obtained from residues of different oilseeds. J. Agric. Food Chem..

[12] Ramadan M.F., Kroh L.W., Maprsel J.T.  (2003). Radical scavenging activity of black cumin (*Nigella sativa* L.), coriander (*Coriandrum sativum* L.), and niger (*Guizotia abyssinica* Cass.) crude seed oils and oil fractions. J. Agric. Food Chem..

[13] Iqbal S., Bhanger M., Anwar F. (2005). Antioxidant properties and components of some commercially available varieties of rice bran in Pakistan. Food Chem..

[14] Iqbal S., Bhanger M., Anwar F.  (2007). Antioxidant properties and components of bran extracts from selected wheat varieties commercially available in Pakistan. LWT-Food Sci. Technol..

[15] Wettasinghe M., Shahidi F. (1997). Antioxidant activity of preformed cooked cured-meat pigment in a [beta]-carotene/linoleate model system. Food Chem..

[16] Zhang H., Chen F., Wang X., Yao H.Y. (2006). Evaluation of antioxidant activity of parsley (*Petroselinum crispum*) essential oil and identification of its antioxidant constituents. Food Res. Int..

[17] Kikuzaki H., Nakatani N. (1993). Antioxidant effects of some ginger constituents. J. Food Sci..

